# A Proposed Clinical Diagnostic Framework for Short Telomere Syndrome

**DOI:** 10.1111/cge.70190

**Published:** 2026-05-31

**Authors:** Andrew Courtwright, Richard A. King, Jennie Vagher, Cate T. Levy, Juan Gallegos F. Orozco, Luis FZ Batista, Afaf Osman, Srinivas Tantavahi, Mary Beth Scholand

**Affiliations:** ^1^ Division of Pulmonary Medicine University of Utah Health Salt Lake City Utah USA; ^2^ University of Utah Health Salt Lake City Utah USA; ^3^ Huntsman Cancer Institute University of Utah Salt Lake City Utah USA; ^4^ Division of Gastroenterology, Hepatology, and Nutrition University of Utah Health Salt Lake City Utah USA; ^5^ Department of Internal Medicine Huntsman Cancer Institute, University of Utah Salt Lake City Utah USA

**Keywords:** bone marrow failure, interstitial lung disease, short telomere syndrome, telomere biology disorder, telomere length

## Abstract

Pathogenic genetic variants in telomere maintenance genes can lead to accelerated telomere length (TL) shortening. Individuals can pass on significantly shortened TL to their offspring, with or without transmission of the causal genetic variant. Carriers and their progeny are at increased risk for disease in organ systems with high life‐time replicative demand, including bone marrow, lungs, and liver. The diagnosis of short telomere related disease is complicated by three factors: the potential for TL inheritance independent of genotype; organ‐specific manifestations that evolve across the lifespan; and the lack of a TL cutoff that reliably predicts phenotypic disease. In this manuscript, we suggest that short telomere syndrome (STS) rather than telomere biology disorder (TBD) is the appropriate term for these diseases. We propose a phenotype‐based approach to STS that distinguishes among four groups: (1) STS (TL < 10th age adjusted percentile in PBMCs with multiple phenotypic manifestations); (2) short telomeres with one phenotypic manifestation; (3) short telomeres without phenotypic manifestations; and (4) multiple phenotypic manifestations without short telomeres. This approach is intended to guide clinical evaluation of patients with short telomeres and longitudinal risk stratification. We also identify research priorities needed to refine diagnostic thresholds and align TL interpretation with disease biology.

## Graphical abstract

We propose a phenotype‐based diagnostic framework for short telomere syndrome that integrates age‐adjusted telomere length, phenotypic manifestations, and genetic findings, where available. The framework is intended to guide clinical evaluation, longitudinal surveillance, and individualized management while acknowledging limitations in current telomere length measurement and evolving genotype–phenotype relationships.

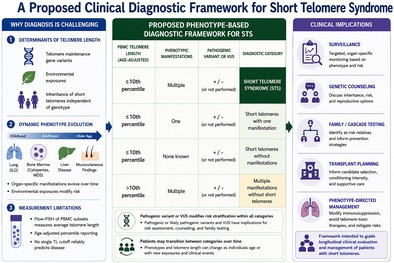

## 1 Introduction

Telomeres are repetitive sequences of DNA at the ends of linear chromosomes. Because a small amount of DNA is lost from the lagging strand with each cell replication, telomeres form a buffer to limit the loss of essential chromosomal genetic information. Telomeric DNA attrition results in telomere shortening over a cell's replicative lifetime [1]. When a telomere reaches a threshold of shortening, a DNA damage response is triggered, leading to cellular senescence or apoptosis [2]. Telomere length (TL) is, therefore, an essential factor in determining a cell's replicative potential. Because telomeres in most cell lines shorten with age, TL is implicated in the pathogenesis of many diseases of aging [3].

For most somatic cells, inherited TL and replicative demand are the key determinants of life‐time replicative potential [4, 5]. Germinal and stem cells, in contrast, express telomerase, a specialized holoenzyme that maintains TL. Pathogenic genetic variants in telomerase genes and/or other telomere maintenance genes can lead to accelerated telomere shortening [6]. Individuals with these variants can pass on significantly shortened TL to their offspring, with or without transmission of the causal genetic variant [7]. Progeny with prematurely shortened telomeres are at increased risk for early onset diseases of aging, particularly in organ systems with high life‐time replicative demand [3]. This includes bone marrow, lungs, and liver, though sufficiently short telomeres can affect most organs.

Depending on the degree of telomere shortening and environmental exposures that increase replicative stress, clinical manifestations of short telomeres may occur across the lifespan [8]. Children with ultrashort telomeres can present with dyskeratosis congenita, a triad that includes bone marrow failure, oral leukoplakia, and reticulated skin pigmentation [9, 10]. Adults with short TL may have one or more manifestations, including early graying of their hair, interstitial lung disease (ILD), non‐cirrhotic portal hypertension, anemia or thrombocytopenia, or myelodysplastic syndrome (MDS) [1, 10].

The diagnosis of short telomere related disease is complicated by three factors: (1) TL inheritance independent of genotype; (2) organ‐specific manifestations that evolve across the lifespan and with environmental exposures; and (3) the lack of a TL cutoff that reliably predicts phenotypic disease. This manuscript considers three central questions: What is the appropriate nomenclature for short telomere related disease? What are the appropriate diagnostic criteria? What technological advances are needed to provide clarity around the relationship between TL and short telomere‐related disease manifestations?

## 2 A Diagnostic Framework

### 2.1 Nomenclature

What term should be used to describe the phenotypic manifestations of abnormal telomere shortening? There are two candidates: telomere biology disorder (TBD) and short telomere syndrome (STS). Both terms have been used in summary reviews, professional society statements, and patient advocacy and consensus guidelines, creating a confusing nomenclature [11, 12, 13]. The phrase TBD captures the idea that organ dysfunction is a result of dysregulation or disorganization in normal telomere biology pathways. In contrast, the term STS emphasizes the role of abnormally reduced TL in causing phenotypic disease. We suggest that STS is the more appropriate label, but that some STS are also TBD.

First, there are disorders of normal telomere function that result in abnormally long telomeres. While there is a movement to label these long telomere syndromes [14] or cancer predisposition syndromes with telomere lengthening [6], the term TBD only speaks to a dysregulation in telomere maintenance, not whether that process results in short or long telomeres. Second, because TL is determined, in part, by parental TL, patients can inherit short or even ultrashort telomeres without genetic variants that impact telomere maintenance function. In these cases, there is no disorder of telomere maintenance, but telomeres are still abnormally shortened. When shortening triggers cellular senescence and/or apoptosis in the setting of increased replicative demand, resulting in organ dysfunction, it is TL that drives phenotypic disease.

Is there a difference between a syndrome and a disorder that should lead us to prefer STS or TBD? Syndromes are classically defined as a constellation of signs or symptoms that occur together in a characteristically recognizable pattern. “Syndrome” is agnostic as to the exact causal agent. For example, acute respiratory distress syndrome is the umbrella term for an inflammatory lung injury with multiple etiologies. In contrast, the term disorder implicitly assumes dysregulation of physiological function, often although not necessarily through a known mechanism. For example, platelet dysfunction disorders are grouped according to the pathway that leads to abnormal platelet activity. In this respect, the term short telomere disorder may be the most accurate categorization of the relationship between cause (short telomeres) and effect (characteristic phenotypic manifestations). Given the clinical confusion the acronym STD would cause, however, retaining STS is the most appropriate choice. This would be akin to other conditions such as Down's syndrome where, despite the identification of a causal mechanism (trisomy 21), the disease is still referred to as a syndrome rather than Down's disorder.

Patients whose short telomeres are a result of pathogenic variants in telomere maintenance pathways or dysregulation of normal telomere maintenance have a STS that is the result of a TBD. In contrast, a patient with preserved telomere maintenance but abnormally shortened telomeres has a STS but no underlying TBD.

### 2.2 Diagnostic Challenges

**FIGURE 1:**
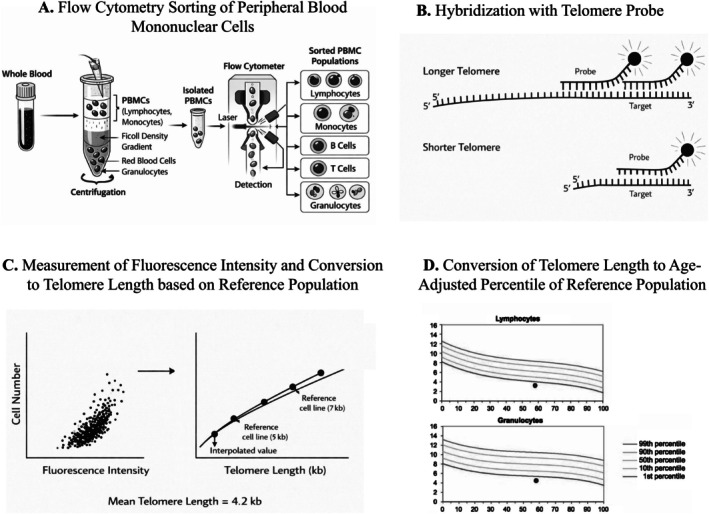
Flow‐FISH measurement of Telomere Length (TL). Peripheral blood mononuclear cells (PBMC) are separated into cell types of interest (A). PBMC are exposed to a fluorescently labelled probe that binds to the telomere sequence (B). Shorter telomeres bind less probe than longer telomeres. The mean fluorescence intensity of bound probe is translated into TL in kilobases (kB) using calibration with cell lines with known TLs (C). The resultant kB measurement reflects the average TL of all counted cells. Because TL shortens with aging, raw TL is converted to a standard deviation of age‐matched controls and reported as a percentile of age‐adjusted TL (D).

Conceptual agreement around the diagnostic criteria for STS remains an ongoing challenge. Some of this difficulty relates to the characteristics of the tests currently used to measure TL in clinical practice. Flow cytometric fluorescence in situ hybridization (flow‐FISH) is generally recognized as the current gold standard for clinical TL measurement [12]. In North America, the only labs that are Clinical Laboratory Improvement Amendment (CLIA) certified to measure TL utilize flow‐FISH (Figure 1A–D). Given that telomeres shorten during aging, raw TL is converted to a standard deviation of age‐matched controls and reported as a percentile of expected TL for age. TL can be measured in various subtypes of PBMC, generating an assessment of TL in, for example, lymphocytes, granulocytes, B cells, and so forth. While limitations of flow‐FISH include the need for fresh cells rather than stored DNA samples, patient and institutional costs, and need for specific expertise to develop reliable flow‐FISH assays for individual centers, flow‐FISH remains the most utilized assay for clinical decision‐making.

Because TL is referenced to age‐matched controls, by definition, 10% of the population will have TL ≤ 10th percentile, 30% will be ≤ 30th percentile, 70% will be ≤ 70th percentile, and so on. In the United States, with a population of 340 million, 34 million people will have TL ≤ 10th age‐adjusted percentile and 3.4 million will be ≤ 1st age‐adjusted percentile. If STS is only defined according to TL ≤ 10th percentile, STS is one of the most prevalent diseases in the United States. More common than chronic obstructive pulmonary disease (11–14 million), STS would challenge chronic kidney disease (33–35 million) and diabetes (39–41 million) as one of the most widespread conditions in America. Even at a cutoff of ≤ 1st percentile age‐adjust TL length, STS would be more common than HIV/AIDS (1–1.5 million), Parkinson's disease (1–1.2 million), or inflammatory bowel diseases (3 million). Defining STS only according to TL cutoff, however, is biologically implausible. This would mean that, in any human population, 10% of people should always be diagnosed with STS, regardless of their absolute TL or whether they have phenotypic manifestations.

The variability of diagnostic approaches is reflected in the current state of the field. For example, in the “Diagnosing Telomere Biology Disordes” chapter of the Team Telomere Diagnosis and Management Guidelines, the focus is on the sensitivity and specificity of lymphocyte TL < 1st percentile in identifying patients with dyskeratosis congenita, using phenotypically unaffected relatives as the control population [13]. In contrast, the recently published International Society of Heart and Lung Transplantation Consensus Statement on Short Telomere Syndrome notes that STS diagnosis requires, at a minimum, PBMC TL ≤ 10th percentile but should also consider the presence of characteristic phenotypic manifestations and genetic testing [12]. And the European Respiratory Society Statement on Familial Pulmonary Fibrosis focuses on a probabilistic framework in which TL < 10th percentile is one of several markers of STS [13]. Influential review articles have focused on associations between TL cutoffs (≤ 10th percentile, < 1st percentile) and outcomes in specific diseases such as pulmonary fibrosis, lung transplant, or bone marrow failure, rather than offering a unified set of diagnostic criteria [8, 15, 16]. And cohort studies have varied significantly in their methodological criteria for a diagnosis of STS, ranging from requiring a pathogenic or likely pathogenic variant and phenotypic manifestations, to lymphocyte TL < 1st percentile and/or a pathogenic or likely pathogenic variant regardless of phenotype, to granulocyte and lymphocyte TL < 10th percentile and multiple phenotypic manifestations, to one phenotypic manifestation and TL ≤ 10th percentile [17, 18, 19].

As the field matures, what is needed is a common diagnostic framework that can orient clinical care around the identification and longitudinal care of patients who have or who may be at risk for phenotypic manifestations arising from short telomeres. It should also integrate the presence or absence of genetic variants (when known) that impact TL, including inheritance patterns and the need for genetic counseling and/or cascade testing in family members without known phenotypic manifestations.

### 2.3 A Four Category Diagnostic Framework

A phenotype‐based diagnostic structure begins with a threshold of PBMC TL as a marker of potential telomere insufficiency, and then distinguishes among four groups: STS with multiple phenotypic manifestations, short telomeres with one phenotypic manifestation, short telomeres without known phenotypic manifestation, and multiple phenotypic manifestations without short telomeres (Table 1). Here, the characteristic phenotypic manifestations may include, but are not limited to, macrocytic anemia or other cytopenias, bone marrow failure, MDS, personal or family history of ILD, oral leukoplakia, non‐cirrhotic portal hypertension, early graying of the hair, reticulated hypopigmentation, nail dystrophy, and early onset clonal hematopoiesis, among others [1]. As the field matures, a structured or tiered approach to phenotype may become warranted. For example, conditions that are paradigmatically associated with critically shortened telomeres—such as bone marrow failure and ILD—may ultimately be weighed more heavily than conditions such as early hair graying, osteoporosis, or emphysema, where the association with pathologic telomere shortening is weaker or less specific.

**TABLE 1:** Phenotype‐based diagnostic scheme for short telomere syndrome.

	Characteristic phenotypic manifestation(s)^a^	PBMC TL	Pathogenic variant or VUS	Example clinical documentation (with and without genetic testing results)
Short telomere syndrome	Multiple	≤ 10th	+/− or not available	–SH is a 47 year‐old with STS (TL < 1st percentile) with ILD, oral leukoplakia, thrombocytopenia, and recurrent SCa of the skin –SH is a 57 year‐old with STS (TL < 1st percentile, pathogenic TERT variant c.2110C>T) with ILD, oral leukoplakia, thrombocytopenia and recurrent SCa of the skin
Short telomeres with one phenotypic manifestation	One	≤ 10th	+/− or not available	–AM is a 62 year‐old with short telomeres (TL < 10th percentile) with IPF –AM is a 62 year‐old with short telomeres (TL < 10th percentile, VUS RTEL1 c.482T>C) with IPF
Short telomeres without phenotypic manifestations	None known	≤ 10th	+/− or not available	–DH is a 21 year‐old with a family history of IPF and personal short telomeres (TL < 1st percentile) without known manifestations of STS –DH is a 21 year‐old with a family history of IPF and personal short telomeres (TL < 1st percentile, pathogenic TINF2 variant c.845G>A) without known manifestations of STS
Multiple phenotypic manifestations without short telomeres	Multiple	> 10th	+/− or not available	–RL is a 4 year‐old without short telomeres (TL > 10th percentile) and multiple STS manifestations (bone marrow failure, gastrointestinal AVMs, oral leukoplakia) –RL is a 4 year‐old without short telomeres (TL > 10th percentile, pathogenic POT1 variant c.965C>T) and multiple STS manifestations (bone marrow failure, gastrointestinal AVMs, oral leukoplakia)

Abbreviations: AVM = arteriovenous malformations; IPF = idiopathic pulmonary fibrosis; PBMC = peripheral blood mononuclear cells; SCa = squamous cell cancer; STS = short telomere syndrome; TL = telomere length; VUS = variant of unknown significance.

^a^ This includes macrocytic anemia or other cytopenias, bone marrow failure, MDS, personal or family history of ILD, oral leukoplakia, non‐cirrhotic portal hypertension, reticulated hypopigmentation, nail dystrophy, early onsent clonal hematopoesis, and early graying of the hair, among others [1].

This structure has several advantages. First, it is simple, requiring only TL and not genetic testing for diagnostic categorization. It captures a central clinical question—has TL reached the threshold at which characteristic phenotypic manifestations of STS have occurred? Given the potential for limited access to genetic testing, insurance coverage barriers, or patient preference not to undergo genetic testing, it is not clinically appropriate to withhold a STS diagnosis from an individual with short telomeres and multiple phenotypic manifestations but no genetic testing. Second, for patients with short telomeres and no known phenotypic manifestations, it invites further investigation (pulmonary function tests, complete blood count, liver function tests, etc.). This also allows longitudinal follow‐up among patients with short telomeres without phenotypic manifestations, acknowledging that they may never develop STS.

Third, although the framework does not require genetic testing or identification of telomere maintenance gene variants, it does not preclude further risk stratification or individualized care plans for individuals who undergo genetic testing and are found to have pathogenic or likely pathogenic genetic variants or variants of uncertain significance (VUS). Incorporating genetic information may be particularly relevant for genetic counseling, cascade testing, family planning, environmental risk factor avoidance (e.g., smoking, excess alcohol consumption, appropriate vaccination, etc.), and surveillance for phenotypic manifestations associated with specific variants. When pathogenic variants or VUS are identified, this information can be integrated into the broader diagnostic category for clinical documentation and individualized longitudinal management (Table 1).

Why distinguish STS (patients with short telomeres and multiple phenotypic manifestations) versus short telomeres with one phenotypic manifestation? First, because the test characteristics of flow‐FISH will always identify 10% of the population as having TL < 10th percentile, there will be some patients who have, for example, IPF and who happen to have TL < 10th percentile. In the absence of any other phenotypic STS manifestations, the relationship between their TL and the pathogenesis of their IPF is not clear. But having short telomeres is still relevant to their IPF management. For example, patients with IPF and TL < 10th percentile who are exposed to immunosuppression have worse survival [20]. And a growing literature suggests that patients with non‐IPF ILD and TL < 10th percentile have worse survival when treated with immunosuppression [21].

Second, patients with short telomeres and multiple phenotypic manifestations (STS) clearly represent a distinct population. For example, IPF patients with TL < 10th percentile and multiple STS manifestations have worse transplant‐free survival than those with TL < 10th percentile and no other STS manifestations [22]. Similarly, patients with ILD and TL < 10th percentile with MDS have a 1 year post‐transplant survival of only 40% compared to 85% among those with ILD alone [23]. Finally, separating patients with one versus multiple phenotypes creates room for patients to change diagnostic categories as they undergo further testing or develop additional phenotypic manifestations. Moving from short telomeres with IPF to STS with IPF, thrombocytopenia, and hepatic fibrosis can orient the patient's clinical care around their subspecialty needs as their phenotypic manifestations are identified or emerge.

Why include a diagnostic category for patients with TL > 10th percentile? This is partly reflective of the current limitations of flow‐FISH technology and the reliance on average TL among a population of PBMC. There are several rare scenarios in which an individual has multiple characteristic phenotypic manifestations of STS but TL significantly above the 10th percentile. First, a patient who has undergone bone marrow transplant for STS‐related bone marrow failure will have the donor's PBMC TL while other somatic cells may reflect “native” shorter TL [24]. Second, somatic gene rescue in the bone marrow can result in PBMC TL that is discordantly longer than other tissues such as the lungs or liver [24]. Third, there are extremely rare variants in telomere maintenance genes (POT1) or associated proteins (Apollo) that result in chromosomal instability whereby the average PBMC TL may fall within age‐adjusted normal percentiles but individual chromosomes have telomeres that are short enough to trigger cellular senesce or apoptosis [25, 26]. It is not known how often this occurs in the absence of these specific variants. While measurement of TL in buccal cells or skin fibroblasts can help identify the first two scenarios, the third remains significantly more difficult.

### 2.4 TL Cutoff

Suppose that a phenotype‐based framework is the most appropriate diagnostic approach within the limitations of currently available TL measurement. Is TL ≤ 10th percentile the appropriate cutoff for considering the diagnosis? Why not < 25th percentile? < 10th percentile? < 5th percentile? ≤ 1st percentile? One difficulty in answering this question is that many cohort studies define TL in relation to other patients in the cohort. So, for example, individuals in the lowest quartile of TL among a cohort of patients with nonalcoholic fatty liver have increased risk of hepatic fibrosis [27]. Similarly, patients in the lowest and middle tertile of TL have increased risk of coronary artery disease compared to the highest tertile [28]. Without use of an age‐adjusted cutoff referenced to a broader population, however, generalizing these results to other cohorts is impossible. At the same time, very few studies report risk ratios or hazard ratios of an outcome using age‐adjusted TL as a continuous variable in a way that allows assessment of inflection points at particular TL cutoffs [21, 29]. Acknowledging that any percentile threshold necessarily dichotomizes a continuous variable and that individuals just above and just below any specific cutoff may have similar risk, the best available current literature suggests that the risk for phenotypic manifestations that impact clinical care decisions starts around the 10th percentile.

For example, cohort studies among patients with ILD, including idiopathic pulmonary fibrosis (IPF) and non‐IPF patterns, TL < 10th percentile is associated with increased risk of disease progression and mortality [30]. As previously noted, patients with IPF and non‐IPF ILD and TL < 10th percentile who are treated with immunosuppression have an increased risk of death [20, 21]. Following lung transplant, patients with ILD and TL < 10th or ≤ 10th are at increased risk for cytopenias requiring adjustment in cell cycle inhibitors [31, 32]. They may also be at risk for large airway complications, cytomegalovirus viremia, and post‐transplant lymphoproliferative disorders [19, 33, 34, 35]. Outside of pulmonology, shorter age‐adjusted TL—often within the lowest decline—has been associated with increased risk of chromosomal mosaicism in aplastic anemia [36]; post‐transplant survival following stem cell transplant for acquired aplastic anemia [37]; cryptogenic liver disease and porto‐sinusoidal vascular disorder [38]; and immune senescence and opportunistic infections [39].

Several caveats remain. First, most studies have evaluated outcomes among patients using percentile cutoffs such as ≤ 10th percentile, and research design may reinforce these thresholds even if telomere biology does not change discretely at that point. Second, most studies are not sufficiently powered to assess whether clinically relevant outcomes are more reliably predicted at a different threshold (like ≤ 5th or ≤ 12th percentile). If risk increases sharply below a lower percentile than ≤ 10th—such as ≤ 5th—grouping patients together at a ≤ 10th threshold may blur relevant distinctions, giving the appearance of risk in the 6th–10th percentile patients that is being driven by those with more significant shortening.

At the current time, adopting the more inclusive but not overly inclusive threshold of ≤ 10th percentile can ensure that ongoing research does not exclude patients at the margin whose clinical and genetic features may ultimately prove relevant. As larger cohorts are developed, future iterations of the diagnostic framework may refine the threshold at which TL becomes clinically relevant or clarify whether TL is best interpreted in the context of specific genetic variants, rates of attrition, or tissue‐specific TL measurement. An inclusive definition at the outset, however, may help support these refinements, knowing that threshold definitions will evolve in response to new data.

### 2.5 Lymphocytes, Granulocytes, or Other PBMC TL?

**FIGURE 2:**
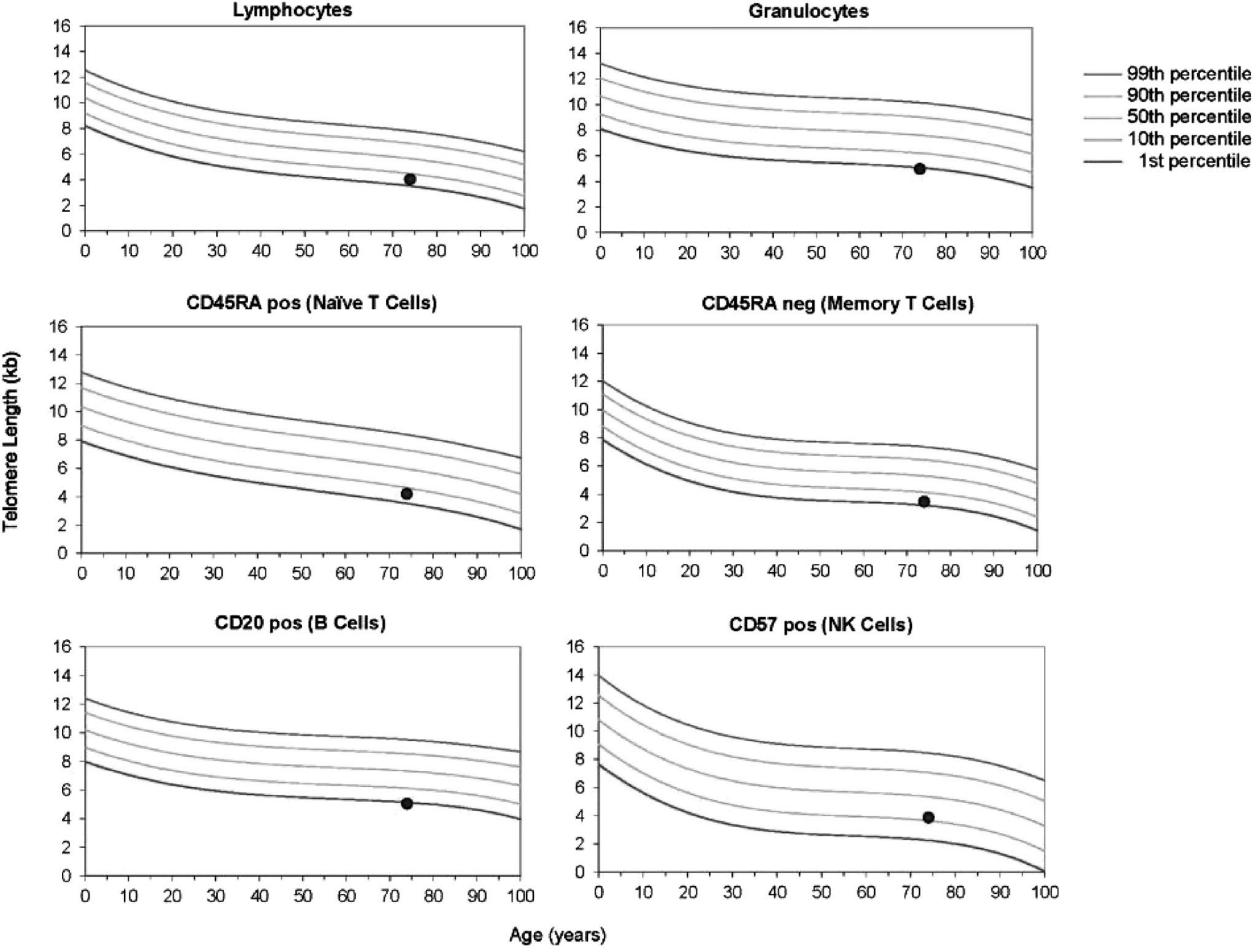
Discordant pattern of age‐adjusted telomere length in tested cell lines.

The final question for any STS diagnostic framework is which PBMC subtype should be used to determine whether TL is ≤ 10th percentile. The most conservative approach would require TL to be ≤ 10th percentile in all PBMC populations tested. While many patients, particularly those with pathogenic variants and multiple phenotypic manifestations will have TL < 1st or ≤ 10th percentile in all cell lines (e.g., lymphocytes and granulocytes or lymphocytes, granulocytes, memory T cells, naïve T cells, NK cells, and B cells, depending on the assay), discordance between cell types can occur. This becomes more common as additional cell populations are measured. One explanation for this phenomenon is inter‐assay variation in test characteristics, resulting in TL that fluctuate slightly above or below the 10th percentile (Figure 2). This is particularly true in older patients. Because these patients have overall shorter telomeres, small differences in probe binding can translate into relatively large shifts in TL percentile.

In such cases, lymphocyte ≤ 10th with other tested cell lines slightly above the 10th percentile threshold would still be consistent with short telomeres, and—when accompanied by multiple characteristic phenotypic manifestations—consistent with STS. By contrast, a patient with lymphocyte TL > 10th percentile, other cell lines at or slightly below the 10th percentile, and characteristic phenotypic manifestations would fall into the category “multiple phenotypic manifestations without short telomeres.” A more challenging scenario arises when one cell line is markedly shortened while the others are relatively preserved. For example, granulocytes in individuals with autoimmune disease and certain hematologic malignancies tend to have more rapid TL shortening. These patients may have TL ≤ 1st percentile in granulocytes alone. In these circumstances, identifying comorbid conditions that selectively influence telomere dynamics in specific cell populations can provide important context for interpreting isolated TL shortening.

Taken together, these observations suggest that although TL ≤ 10th percentile across all tested cell populations provides the greatest diagnostic clarity, interpretation should integrate TL across individual PBMC subsets—particularly lymphocytes—alongside the patient's phenotypic manifestations. In this case, lymphocytes are preferred because they reflect replicative demand in long‐lived hematopoietic populations and are less susceptible than granulocytes to transient shifts associated with inflammatory or myeloproliferative processes.

### 2.6 Implications for Clinical Care and Remaining Diagnostic Challenges

Our framework is intended to standardize clinical decision making across the four diagnostic groups. For patients with STS, evaluation should prioritize comprehensive multi‐organ assessment and early subspecialty co‐management among clinicians with experience caring for STS patients. In contrast to, for example, ILD guidelines, which require multidisciplinary consensus for the *classification* of a particular ILD subtype, the proposed STS diagnostic framework does not require such consensus. Rather, the aim is to have a multidisciplinary group available to navigate organ‐specific phenotypic manifestations. This includes decision‐making about transplant referral, immunosuppression intensity, infectious prophylaxis, bone density testing, and cancer screening, all of which should be informed by the relevant telomere biology. For patients with short telomeres and a single phenotypic manifestation, management should include phenotype‐directed care but with consideration of treatment thresholds (such as use of immunosuppression in ILD) modified by the presence of short telomeres. Longitudinal evaluation of these patients should include assessment for subclinical manifestation of other telomere‐related organ dysfunction. For patients with short telomeres and no phenotypic manifestations, particularly younger individuals, the framework supports longitudinal surveillance and baseline screening (complete blood count, pulmonary function testing, liver function testing, etc.) and informing decisions about genetic counseling and family planning. Finally, for patients with multiple phenotypic manifestations but TL > 10th percentile, management should proceed as with STS, with consideration of TL testing in alternative tissue or evaluation for somatic rescue of bone marrow. Across all diagnostic categories, referral to genetic counselors should be considered to guide interpretation of TL and variant testing, assess heritable risk, and coordinate cascade testing, where appropriate.

Given the complexity of the genotype–phenotype relationship, the chronological evolution of characteristic phenotypic manifestations, and currently available telomere measurement tools, there will be gray areas and ongoing clinical challenges with any diagnostic framework. Tables 2, 3 highlight several of these scenarios, including insufficient quantity of PBMC for TL analysis, telomere maintenance gene variants detected in the setting of somatic clonal hematopoiesis, and pathogenic germline variants in telomere maintenance genes but normal PBMC TL [40] (Table 2 and Table 3). The latter scenario remains a particular challenge for any STS or TBD diagnostic framework. Some individuals with pathogenic variants in PARN may have normal TL and no phenotypic manifestations [40]. In other cases, individuals with pathogenic variants in telomere maintenance genes may not yet demonstrate substantial PBMC shortening, may exhibit incomplete penetrance, or may have somatic rescue of bone marrow progenitor cells, resulting in apparently normal PBMC TL despite shortened telomeres in other tissues. Given the current state of the field and the evolving nature of TL testing, we favor developing individual care plans for patients in these scenarios rather than creating an additional diagnostic subcategory defined solely by the presence of a pathogenic variant with normal PBMC TL and absent phenotypic manifestations.

**TABLE 2:** Diagnostic challenges related to telomere length measurement.

Scenario	Causes	Management options
Insufficient quantity of peripheral blood mononuclear cells for analysis	Telomere length testing is performed while the patient is on medications that affect lymphocyte count (e.g., rituximab, anti‐thymocyte globulin, chemotherapy)	Repeat testing after lymphocyte count has recovered Consider testing on alternative tissue source (skin fibroblasts)
Distinct peaks detected for telomere length distribution within a cell subset	Proliferative pressure on subpopulation with the shortest telomere length. Immune exhausted cell Clonal population	Careful assessment of phenotypic manifestations and genetic test results Evaluate for autoimmune disease and clonal hematologic disorder
Inability to evaluate the patient's own lymphocyte telomere length	The patient has had a bone marrow transplant prior to checking telomere length, so lymphocytes in blood are donor's lymphocytes	Consider testing using an alternative tissue source (skin fibroblasts)
Telomere lengths are > 10th age adjusted percentile in all cell lines but the patient has multiple phenotypic manifestations of short telomere syndrome	Measurement of mean telomere length fails to capture cell populations (or individual chromosomal telomere lengths) with critically shortened telomeres Somatic rescue of bone marrow leads to normal telomere length in peripheral blood mononuclear cells	Consider telomere length evaluation using Telomere Shortest Length Assay (TeSLA) or evaluation for variants (POT1, APOLLO) associated with unstable telomeres but normal mean telomere length Consider testing using an alternative tissue source (skin fibroblasts)

**TABLE 3:** Diagnostic challenges in specific clinical scenarios.

Scenario	Example	Causes	Management options
Uncertainty whether the phenotype is connected to short telomeres	42 year‐old male with acquired aplastic anemia with PNH clone detected, telomere length 1‐10th percentile, no genetic testing	The PNH clone is highly suggestive of an acquired rather than hereditary cause of the aplastic anemia	Genetic testing Evaluate for other phenotypic manifestations If bone marrow transplant is planned, consider managing like a short telomere syndrome
Uncertainty about penetrance	64 year‐old male with heterozygous TERT variant on hereditary cancer panel from blood sample, but normal telomere length testing and no phenotypic manifestations	Since the genetic testing was performed on blood cells, there is a chance the TERT variant is a somatic clonal hematopoiesis variant rather than a germline variant	Rule out clonal hematopoiesis by genetic testing on skin fibroblast sample If germline variant is confirmed, perform baseline evaluation to assess for other phenotypic manifestations and consider checking telomere length in skin fibroblasts
Discordant telomere length in cell subsets	70 year‐old female with lymphocyte TL > 10th percentile, other cell lines at or slightly below the 10th percentile, and characteristic phenotypic manifestations	This patient falls into the category “multiple phenotypic manifestations without short telomeres”	Manage similarly to a patient with short telomere syndrome
Short telomeres are isolated to granulocytes	25 year‐old female with aplastic anemia and TL < 1st percentile in granulocytes, but > 10th percentile in lymphocytes	This likely represents acquired telomere shortening in granulocytes rather than a hereditary short telomere syndrome. This can occur in certain hematologic malignancies and autoimmune disease	No further short telomere evaluation unless other phenotypic manifestations develop
No pathogenic variant and no phenotypic manifestations but strong family history	16 year‐old male with paternal pathogenic TERT variant and multiple affected first degree relatives. Genetic testing is negative for pathogenic TERT variant	Because critically shortened telomeres can still be inherited, even in the absence of a pathogenic variant, telomere length testing should still be performed	If telomere length testing reveals TL < 10th percentile, follow for longitudinal manifestations of short telomere related disease
Pathogenic germline variant in telomere maintenance gene but normal telomere length	44 year‐old with family history of interstitial lung disease and pathogenic PARN variant. TL at 50th percentile in all cell lines.	Somatic rescue of bone marrow progenitor cell telomere length Incomplete penetrance	Evaluate for other phenotypic manifestations Consider testing using an alternative tissue source (skin fibroblasts) Consider genetic testing for TERT promoter variants or other variants associated with somatic rescue

## 3 What is Needed Next

A central difficulty for any diagnostic scheme is the reliance on a TL assay that is reported as an age‐adjusted percentile. The inclusion of phenotype is important but does not bridge the mechanistic gap between whether a patient happens to have TL in the 10th age adjusted percentile and a specific STS manifestation (like ILD) or whether short TL causes or significantly contributes to that manifestation. Furthermore, mean TL is merely a surrogate for the proportion of senescent cells, which can be affected by assay variability, immune cell subset composition, decreasing sensitivity with age, and genetic and environmental determinants of telomere maintenance, and may not mirror telomere dysfunction in other tissues.

To address these limitations and further refine STS diagnostic criteria, we believe there are four research priorities. First, there is a need for clinically scalable methods to measure the shortest chromosomal TL in PBMC rather than median or average TL. While such technology has been developed—for example, telomere shortest length assay (TeSLA) and single telomere length analysis (STeLA)—it is labor intensive and subject to significant implementation and optimization phase laboratory training [41]. High throughput technology using nanopore or long read sequence may bridge this gap, although clinical applications remain distant [40].

Second, in vitro studies using pluripotent stem cells and lung organoids or related tissue constructs can better define the point at which absolute TL shortening triggers/is associated with cellular senescence and phenotypic disease [15, 42, 43]. Third, studies are needed to better correlate PBMC TL and tissue TL. Because of acceleration in TL attrition in the setting of environmental exposures (cytotoxic medications or smoking in lung disease, hepatotoxic medications or alcohol in liver disease), understanding to what extent PBMC is an appropriate surrogate for tissue TL in patients with advanced lung or liver disease can help better capture when these conditions are telomere‐related [44].

Finally, large, multicenter longitudinal cohorts are needed to examine age‐adjust TL cutoffs as a continuous variable and identify clinically meaningful inflection points below (or above) the 10th percentile. While individual cases would still require clinical judgment, current studies, which are typically between 40 and 200 patients, lack the statistical power to explore granular TL cutoffs [21, 22, 23]. Large cohorts could also address the clinical relevance of discordant TL results (such as lymphocytes ≤ 10 percentile but granulocytes > 10th percentile) and whether there are prognostic differences between patients with pathogenic variants and variants awaiting requiring further classification.

## 4 Conclusions

In this manuscript, we have argued that STS should be the preferred term for disease manifestations arising from pathologic telomere shortening, while recognizing that TBDs represent a subset of STS attributable to pathogenic variants in telomere maintenance genes. We have proposed that a phenotype‐based diagnostic framework in which STS is reserved for patients with PBMC TL ≤ 10th percentile and multiple characteristic phenotypic manifestations, while distinguishing patients with TL ≤ 10th percentile and one or no known phenotypic manifestations. This structure is intended to orient clinical evaluation and longitudinal surveillance around phenotypes, while preserving the ability to incorporate genetic findings. This diagnostic framework focuses clinical care on phenotype, including encouraging examination for other organ dysfunction associated with TL ≤ 10th percentile and making treatment decisions utilizing the available research around patients with TL ≤ 10th, such as in ILD. While some areas of diagnostic uncertainty will persist within our proposed nomenclature, it also points to areas of needed technologic development and epidemiologic research to further refine the diagnosis and care of patients with STS.

## Author Contributions

All authors contributed to manuscript design, critical revision, and final approval. A.C. participated in the initial draft of the paper and the figures. A.C., R.K., and J.V. participated in the drafting and revisions of the tables.

## Funding

The authors have nothing to report.

## Conflicts of Interest

The authors declare no conflicts of interest.

## Data Availability

Data sharing not applicable to this article as no datasets were generated or analysed during the current study.
